# The role of soluble fibrin during anticoagulant therapy: a case report

**DOI:** 10.1186/s12959-015-0053-1

**Published:** 2015-07-06

**Authors:** Tohru Kawakami, Nobukiyo Tanaka, Hiroki Ishihara, Hiroyoshi Ohno

**Affiliations:** Division of Laboratory Medicine, Ichinomiyanishi Hospital, 1 Kaimei-hira, Ichinomiya, Aichi 494-0001 Japan; Division of Cardiology, Ichinomiyanishi Hospital, 1 Kaimei-hira, Ichinomiya, Aichi 494-0001 Japan

**Keywords:** Soluble fibrin, Pulmonary thromboembolism, Atrial fibrillation, Deep vein thrombosis, Warfarin, Apixaban, Dabigatran

## Abstract

Warfarin, dabigatran, and apixaban are used for preventing ischemic stroke due to non-valvular atrial fibrillation (NVAF). However, it is often challenging to select the appropriate anticoagulant. We present the case of a 70-year-old male patient with persistent NVAF who developed pulmonary thromboembolism (PTE), deep vein thrombosis (DVT), and left atrial thrombus during anticoagulant therapy with warfarin. Intravenous recombinant tissue plasminogen activator was administered during his acute PTE. Heparin and apixaban were administered over 28 days; heparin was discontinued after the DVT resolved, while apixaban was administered to prevent ischemic stroke. Two days after heparin was discontinued, the patient experienced an ischemic stroke. Dabigatran was administered for secondary ischemic stroke prevention. Soluble fibrin (SF) levels remained elevated during treatment with heparin and apixaban and returned to normal after apixaban was replaced with dabigatran. Monitoring of SF may be useful as an index for selection of anticoagulants.

## Background

Left atrial appendage (LAA) thrombus is commonly associated with non-valvular atrial fibrillation (NVAF) and causes thromboembolic complications. Warfarin is used not only for prevention of thromboembolic complications in NVAF patients, but also for treatment of deep vein thrombosis (DVT) [1, 2]. The optimal therapeutic range for anticoagulation with warfarin to prevent thromboembolic events in NVAF patients extends over INR values of 2.0 to 3.0 [3, 4]. Recently, non-vitamin K antagonist oral anticoagulants (NOACs) have been used for preventing ischemic stroke due to NVAF. These drugs are superior to warfarin in preventing stroke or systemic embolism and cause less bleeding [5, 6]. An index of the effect of NOACs has not been established, and the challenge to physicians is how to select an appropriate anticoagulant from this group of drugs.

Recent studies have shown that soluble fibrin (SF) and D-dimer levels have been considered useful for the diagnosis of thrombosis. Soluble fibrin is generated from thrombin-fibrinogen reactions and is a potentially useful marker of hypercoagulability, while D-dimer is a small protein fragment and is detected only after a blood clot is degraded by fibrinolysis [7–12].

## Case presentation

A 70-year-old male underwent warfarin treatment to prevent the thromboembolic complications of NVAF. He consulted our hospital due to severe dyspnea and hypotension. His warfarin treatment time in therapeutic range (TTR) was 100 % during the 3 months prior to hospitalization in our institution.

**Fig. 1:**
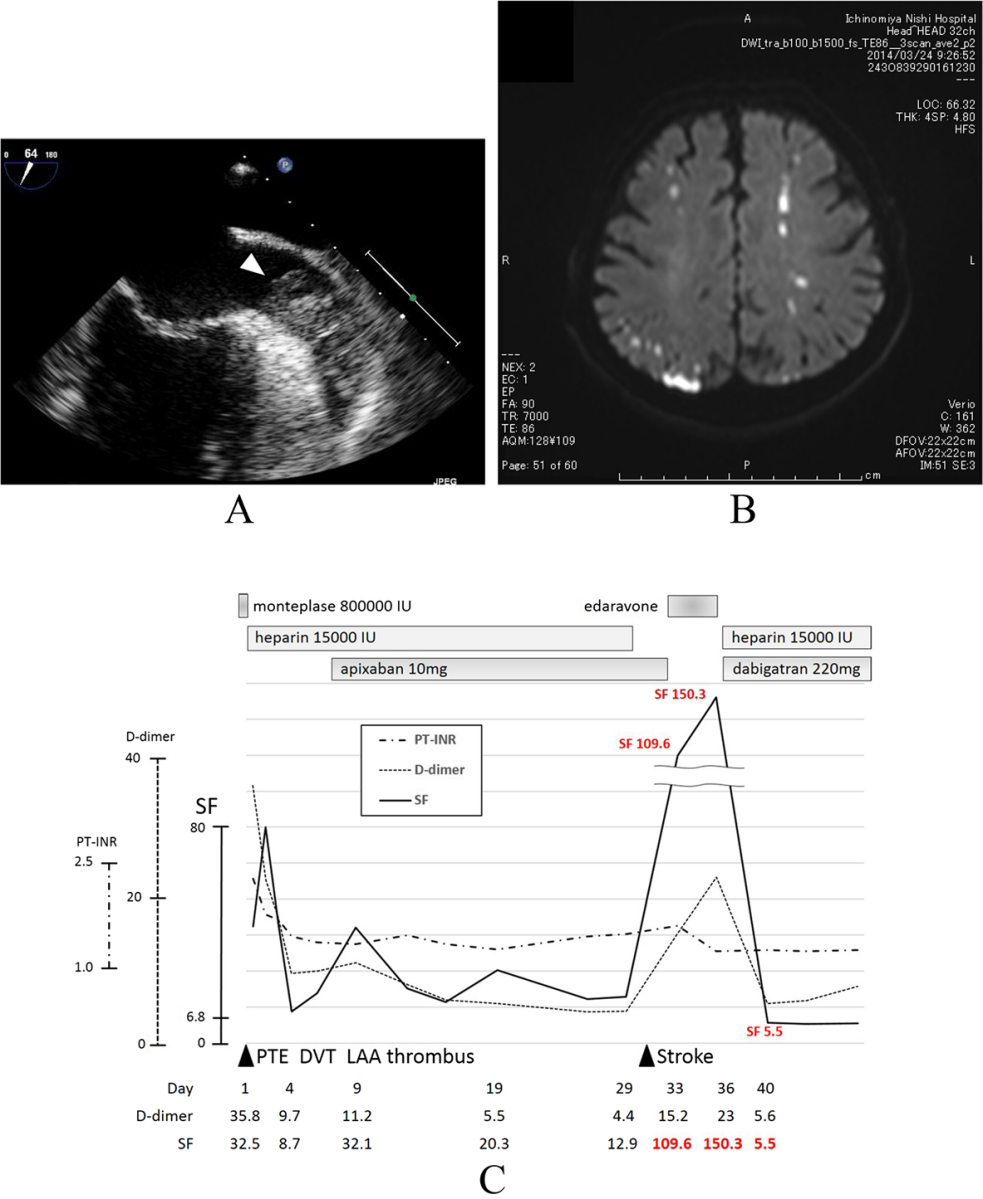
Transesophageal echocardiography, MRI, laboratory data for the coagulation system and administration of medicine. **a** Thrombus formation (arrow) in the apex of the left atrial appendage (LAA) on Day 8. **b** Acute multiple ischemic stroke due to NVAF in MRI (T2-weighted image). **c** Plot of laboratory coagulation data demonstrates high levels of SF during apixaban and heparin therapy, and a decrease in SF starting after dabigatran and heparin therapy

His pulmonary thromboembolism (PTE) and DVT were revealed by enhanced computed tomography (CT). He underwent thrombolysis with 80,000 IU of tissue plasminogen activator (t-PA). At hospitalization, he had elevated levels of SF (32.5 μg/mL) and D-dimer (35.8 μg/mL), with an international normalized ratio (INR) within his target therapy level (2.28), normal levels of anti cardiolipin beta2-glycoprotein I complex antibody (<0.7 U/mL) and protein C activity (98 %), and decreased protein S activity (46 %). His CT findings suggested lung cancer and LAA thrombus. After thrombolysis with t-PA, we recognized that warfarin therapy was not available for this patient, and we started apixaban therapy (5 mg twice daily) combined with heparin (15,000 IU). We performed transesophageal echocardiography, which revealed thrombus formation in the LAA (Fig. 1a). The elevated SF and D-dimer levels decreased, but did not return to normal during treatment with apixaban and heparin.

At Day 29 of hospitalization, resolution of his DVT was confirmed with echo-venography. We discontinued heparin dosing, but continued apixaban treatment. At Day 31, the patient noted an abnormal feeling in his right hand, this symptom continued over time. At Day 33, multiple ischemic strokes were revealed using magnetic resonance imaging (Fig. 1b). Apixaban therapy was discontinued and edaravon 60 mg was started. By Day 36, the patient’s SF and D-dimer levels were significantly elevated. We started dabigatran therapy (110 mg twice daily) with heparin (15,000 IU) for secondary prevention of embolism. At Day 40, his SF level had normalized (5.6 μg/mL) and his D-dimer level decreased (5.5 μg/mL) (Fig. 1c). The patient did not have a recurrence of thrombosis after dabigatran therapy with heparin, and chemotherapy (CBDCA + PEM) was started to treat his lung cancer (adenocarcinoma, T4NXM1a, stage IV) at Day 65. However, the patient expired after 79 days of hospitalization.

Note: All blood samples were collected before breakfast. During this trough period, INR, prothrombin time, SF, and D-dimer were all detectable. All assays were performed in our institute’s laboratory. For quantitative determination of coagulation tests, an automated coagulation analyzer was used (Coapresta 2000; Sekisui Medical Co., Ltd., Tokyo, Japan). Prothrombin time was measured with Coagpia PT-N (Sekisui Medical Co., Ltd.), whose international sensitivity index is almost equal to 1.0. Sodium citrate-preserved plasma was used to measure SF (Nanopia SF; Sekisui Medical Co., Ltd.) and D-dimer (Nanopia D-dimer; Sekisui Medical Co., Ltd.). Processing and analysis of all samples was performed within 90 min of sample collection. This case report was approved by the ethics committee of ichinomiyanishi hosiptal.

## Discussion

The purpose of anticoagulant therapy is prevention of thrombosis formation. In this case, warfarin therapy with suitable TTR did not prevent thrombosis. The optimal therapeutic range for anticoagulation with warfarin in the prevention of thromboembolic events with NVAF patients extends over INR values of 2.0 to 3.0. It has been reported that major bleeding is observed only at INR values above 2.5 on anticoagulation with warfarin [3]. Warfarin therapy is evaluated by monitoring of INR level; the measurement of INR value during warfarin therapy is a suitable tool for assessment of bleeding risk, but it does not alert the physician to hypercoagulability. Apixaban and dabigatran are superior to warfarin in preventing stroke or systemic embolism, and cause less bleeding [5, 6].

Soluble fibrin is generated from thrombin-fibrinogen reactions, and high SF levels indicate hypercoagulability [13–15]. In a previous report, SF and D-dimer levels decreased along with LAA thrombus resolution without prolongation of INR when the patient was under apixaban therapy [16]. In this case, it was observed that SF and D-dimer levels were elevated at hospitalization and apixaban therapy with heparin could not reduce the SF level to normal. The patient developed Ischemic stroke under these conditions. After starting dabigatran therapy with heparin, his SF level returned to normal. For this patient, dabigatran therapy with heparin might have been more effective for inhibiting hypercoagulability than apixaban therapy with heparin. Elevated SF levels may indicate hypercoagulability during anticoagulation therapy; if a patient’s SF level was elevated during anticoagulation therapy, it may indicate the necessity of selecting a different anticoagulant agent.

We thought that hypercoagulability of this patient was due to his lung cancer. When NVAF patients developed malignancy, it is necessary to maintain strict anticoagulation therapy against hypercoagulability to aid in preventing thrombosis [17, 18].

## Conclusions

We report the case of a patient with NVAF-emergent PTE, DVT, and LAA thrombus during warfarin therapy, with subsequent ischemic stroke during apixaban therapy. The patient’s SF level increased despite anticoagulation therapy. Monitoring SF values may be effective in evaluation of the hypercoagulable condition in anticoagulant therapy, and may serve as an index for changing anticoagulants.

## Abbreviations

LAA: Left atrial appendage
NVAF: Non-valvular atrial fibrillation
PTE: Pulmonary thromboembolism
DVT: Deep vein thrombosis
NOACs: Non-vitamin K antantagonist oral anticoagulants
SF: Soluble fibrin
TTR: Time in therapeutic range
CT: Computed tomography
t-PA: Tissue plasminogen activator
INR: International normalized ratio
PT: Prothrombin time
